# Research on time series characteristics of the gas drainage evaluation index based on lasso regression

**DOI:** 10.1038/s41598-021-00210-z

**Published:** 2021-10-18

**Authors:** Shuang Song, Shugang Li, Tianjun Zhang, Li Ma, Lei Zhang, Shaobo Pan

**Affiliations:** 1 College of Energy, Xi’an University of Science and Technology, No. 58, Yanta Middle Road, Beilin District, Xi’an City, 710054 Shaanxi Province China; 2 College of Safety Science and Engineering, Xi’an University of Science and Technology, No. 58, Yanta Middle Road, Beilin District, Xi’an City, 710054 Shaanxi Province China; 3 College of Communication and Information Engineering, Xi’an University of Science and Technology, Xi’an, 710054 China

**Keywords:** Energy science and technology, Engineering

## Abstract

The evaluation of the coal mine gas drainage effect is affected by many factors, such as flow rate, wind speed, drainage negative pressure, concentration, and temperature. This paper starts from actual coal mine production monitoring data and based on the lasso regression algorithm, features selection of multiple parameters of the preprocessed gas concentration time series to construct gas concentration feature selection based on the algorithm. The three-time smoothing index method is used to fill in the missing values. Aiming at the problem of different dimensions in the gas concentration time series, the MinMaxScaler method is used to normalize the data. The lasso regression algorithm is used to perform feature selection on the multivariable gas concentration time series, and the gas concentration time series selected by the lasso feature and the gas concentration time series without feature selection are input. The performance of the ANN algorithm for gas concentration prediction is compared and analyzed. The optimal α value and L1 norm are selected based on the grid search method to determine the strong explanatory gas concentration time series feature set of the working face, and an experimental comparison of the gas concentration prediction results before and after the lasso feature selection is performed. We verify the effectiveness of the algorithm.

## Introduction

China is the world’s largest coal producer and consumer. Among the primary energy resources in the production and consumption of China, coal accounts for as high as much as 77% and 66%, respectively. In recent years, in the process of coal exploitation, the frequency of gas accidents has been decreasing year by year, but among all kinds of accidents, the casualty rate of gas accidents is higher than that of all accidents^1^. During the process of gas control, gas drainage is an effective measure of gas control; nevertheless, gas drainage is often affected by the gas concentration; therefore, elucidating the change law of gas concentration can effectively address the problem of drainage, reduce the incidence of gas accidents and improve the management level of safety products in the process of coal mining^2,3^.

Wang et al.^4^ adopts the Laida criterion to deal with missing values. It builds a Larange-ARIMA real-time prediction model for coal and gas outburst based on the ARIMA algorithm and the L1 minimization principle. This model improves the utilization of monitoring information. Han et al.^5^ proposed a method of using the Markov model to correct the prediction results of a third-order gray neural network model, used the method to analyze and predict gas concentrations in different locations of a coal mine at different times, and solved the problem of low accuracy of prediction values while using a gray neural network for gas concentration prediction. Wu et al.^6^ used a method based on fuzzy information granulation, a support vector machine (SVM), and a differential evolution algorithm (DE) to establish a prediction model. Residual correction was carried out according to the Markov chain and predicted the trend of gas; therefore, the prediction performance of gas concentration was greatly improved. In addition, other experts and scholars have used numerous methods to predict the gas concentration. Among them, Zhao^7^ extracted the nonlinear high-frequency fluctuation term and low-frequency trend term in the gas concentration series by using empirical mode decomposition (EMD).Then, a limit learning machine (ELM), which is different from each component, was constructed to track and forecast, the gas concentration Prediction of the superposition of each component was found to useful in determining the gas concentration, A gas concentration time series prediction model based on EMD and an ELM was constructed, with significance for effective control and early warning of gas concentration over a limit problem. A real-time prediction system of gas concentration based on streaming linear regression, which uses the real-time streaming data processing framework spark streaming, was constructed by Wu et al.^8^. Yang et al.^9^ proposed a prediction model of multivariate time series based on the multivariate distribution lag model. In addition, adjusted group lasso technique was used to extract the characteristics of the gas concentration time series. Then, the generalized cross-validation (GCV) method was used to find the best parameter combination in the model, and the combination of parameter variables was realized through grouping optimization. The time series of gas concentration after the screening was taken as input, to improve the accuracy of gas concentration prediction. Zhao^10^ reconstructed the phase space of gas concentration time series based on chaos theory and individually determined the time delay and embedding dimension of the phase space. In phase space, the weighted first-order local method was used to establish the prediction model of the gas concentration in the working face, effectively improving prediction accuracy. In the process of modeling, Wang et al.^11^ converted the raw data to fuzzy data, and the model of a fuzzy coefficient was transformed into solving linear programming. To avoid misevaluation caused by the inaccuracy of the prediction value of the specific result, a fuzzy polynomial prediction based on time was proposed. Yang and Zhou^12^ describeds the hourly variation in daily gas concentration, by mapping its time series into polar coordinates to create an elliptic orbit trace for further analysis, and proposed an elliptic orbit model, to address the problem about of modeling and predicting gas concentration variation.

Although the above related algorithms have been applied to different degrees in practice, they still have some limitations. The model of gas concentration prediction based on gas production monitoring data focuses mainly on time series analysis. Specifically, the existing problems mainly include the following: ① Numerous methods use only a univariate model to predict the gas concentration, without considering the influence of temperature, wind velocity, negative pressure, gas flow or other factors on the gas concentration; ② considering the multivariable time series of gas concentration, the method relies only on the artificial method for feature selection, which fails to remove the feature variables with weak correlation and retains the features with strong correlation; and ③ thehe data sample of the training model is small, the time span is short, and the applicability of the training model in the actual application scenarios is weak. Therefore, to ensure the accuracy and robustness of gas concentration prediction, the gas concentration prediction method still needs to be further studied.

## Discussion

The prediction accuracy of the two feature sets selected by the lasso feature was compared to verify the effectiveness of the gas concentration time series feature selection method based on lasso regression in this paper. The feature set selected by the lasso feature predicted the gas concentration in the working face better than the previous method.

The prediction result using this method had better prediction accuracy.

### Dataset division

In this experiment, the commonly used 7:3 dataset division method was used to divide the processed data into a training set and a test set. The training set was used to train the prediction algorithm, and the test set was used to test the learning effect of the prediction model. In this experiment, the collected data from the coal mine safety production monitoring system were selected. According to the abovementioned dataset division method, the training set contained 7000 pieces of data and the test set contained 3000 pieces of data. The Fifty pieces of data were separately reserved to verify the predictive ability of the model.

### Determine the two feature sets to be compared for prediction accuracy

To verify the reliability of the lasso feature selection, this paper includes the original gas concentration time series of 9 variables and the gas concentration time series selected by the features of the lasso method, which are divided into two feature variable sets. The two feature variable sets are denoted as sets I and II and used as the input variable of the predictive model. The 9 variables include the negative pressure of drainage, return air flow gas concentration, mixed flow, face air flow, upper corner gas concentration, pure flow, temperature, cumulative drainage daily flow and drainage concentration. The gas concentration time series selected by the features of the lasso method includes the return air flow gas concentration, the upper corner gas concentration and the temperature.

### Experimental parameter settings

The experiment used the ANN algorithm as the prediction model to compare and analyze the prediction performance of the algorithm before and after lasso feature selection. The structure of the ANN after grid search was set as: 2 hidden layers, 12 hidden layer neural units, a batch size of 20, and a learning efficiency of 0.001.

### Comparison of the fitting effect of two feature sets of the input to the ANN

**Figure 1:**
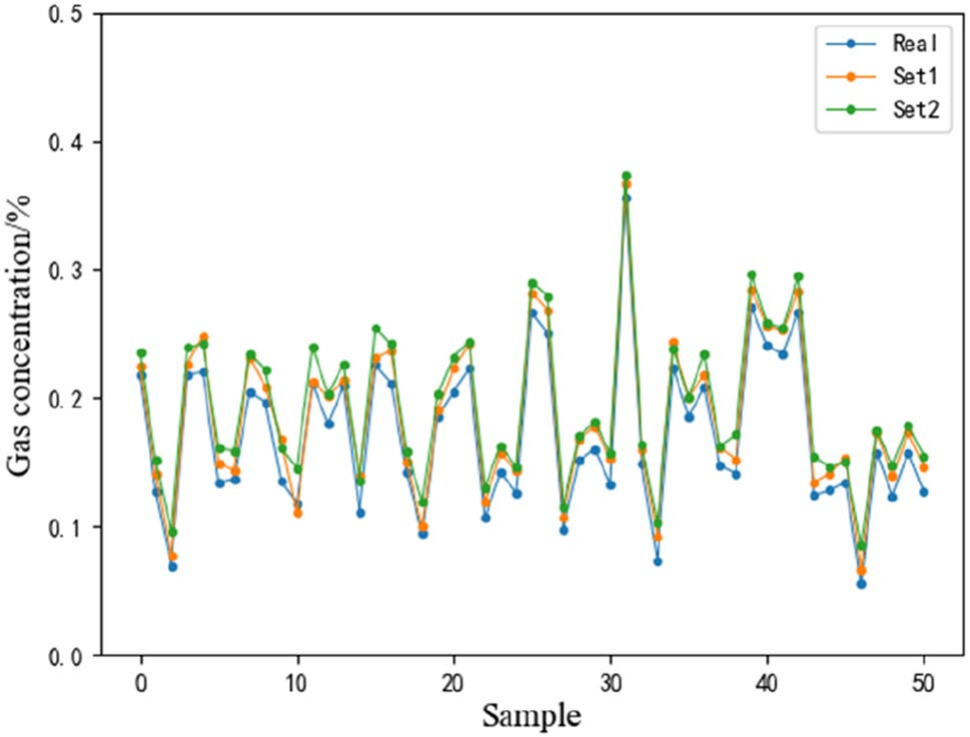
Fitting graph of prediction results of gas concentration in the working face based on the ANN.

The fitting effect of two different feature sets the input to the ANN is shown in Fig. 1.

The time series of gas concentration without feature selection (set I: suction negative pressure, return air flow gas concentration, mixed flow, working face air volume, upper corner gas concentration, pure flow, temperature, cumulative daily drainage flow and drainage concentration) as the input prediction error was greater than the gas concentration time series selected by the lasso feature (set II: return air flow gas concentration, upper corner gas concentration and temperature) as the input prediction error. The fitting effect of gas concentration time series is better. Especially at the inflection point of the gas concentration time series, the prediction error is lower and the fitting effect is better.

**Figure 2:**
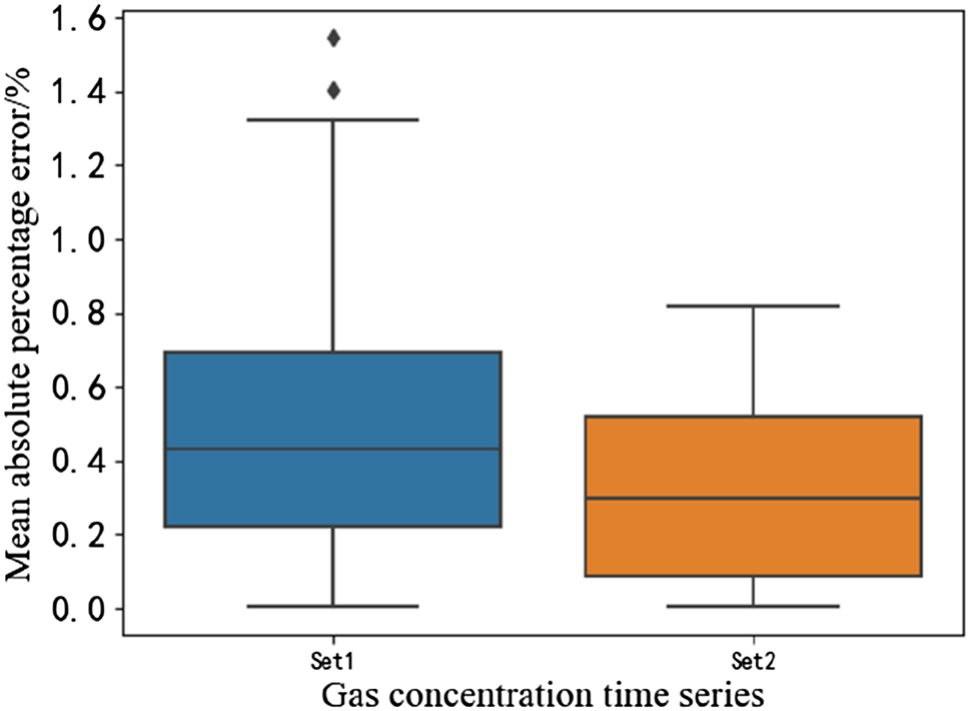
Prediction result error box plot.

The performance of the prediction MAPE with set II as input was notably better than that with set I as input, as shown in Fig. 2. The prediction performance of the MAPE was stable, with few large error points. The main concentration interval of MAPE is more concentrated and low, and the prediction accuracy is higher. The comparison table of ANN prediction performance with different variable sets as input is shown in Table 1. The prediction performance with set II as input was more stable, and the MAPE could be reduced to 0.3274. The prediction performance was more reliable, the MAPE median could be reduced to 0.3625, and R2 could be increased to 0.8852.

**Table 1:** Comparison of ANN prediction performance with different variable sets as input.

Performance	MAPE	MAPE median	R^2^
Set I	0.5074	0.4213	0.8091
Set II	0.3274	0.3625	0.8852

### Experimental result

The experimental results found that the lasso method could obtain a more correlated gas concentration time series than other methods. The lasso method used $\alpha$ optimization and L1 absolute value regularization method for variable screening, which could effectively remove variables that were less correlated with the target variable in the gas concentration time series. In the same time, the prediction model that took the gas concentration time series after lasso feature selection as input could effectively improve the accuracy of gas concentration prediction. The gas concentration time series after feature selection by the Lasso method can effectively remove the feature variables that are less relevant to the target variable in the gas concentration time series, and retain the variables that are more relevant to the target variable. Effectively improve the interpretability of the gas concentration time series. As Lasso compresses feature variables with less correlation through the regression regular method, as to remove redundant variables. Compared with the gas concentration time series without feature selection, the gas concentration time series after Lasso feature selection is used as input. An ANN model is used to predict the gas concentration at the working face, which can effectively improve the accuracy of the prediction.

## Methods

To ensure the accuracy and authenticity of model prediction, this experiment takes the production monitoring time series data of July 1, 2020 solstice and November 2, 2020, working face as the training sample, with a total of 10,000 pieces of data, including the drainage from negative pressure/kPa (X_1_), gas concentration in return air/% (X_2_), mixture flow rate/m^3^/min (X_3_), airflow of working face m^3^/min (X_4_), gas concentration at upper corner angle/% (X_5_), drainage pure flow/m^3^/min (X_6_), temperature/°C (X_7_), the daily and cumulative extraction volume m^3^/min (X_8_), extraction concentration /% (X_9_), gas concentration in working face /% (X_10_).

### Preprocessing of time series data of evaluation index for gas drainage

The data quality has an important influence on the accuracy of the prediction model. The data acquisition equipment and storage medium are susceptible to the influence of nonhuman factors in the process of data transmission, resulting in the absence of data and data anomalies in the raw data. Therefore, in this experiment, the raw data were cleaned twice successively.

#### Outlier correction based on the near average value method

The usual ways to deal with outliers in the raw data are as follows: the data samples containing abnormal data were directly deleted, the abnormal samples were treated as missing values, the abnormal samples were not processed, and the average value method was adopted for correction. However direct use of untreated outliers leads to an increase in sample variance and reduces the prediction accuracy of the model, and deleting samples with outliers directly loses important information. Since the outliers accounted for a small proportion of the sample data of the gas concentration time series adopted in this experiment and there were no continuous outliers, the near average value method was adopted to correct the outliers without considering the influencing factors among various features^13,14^. The detailed operation principle is shown in Eq. (1).

1 $$ x_{t} = \frac{{x_{t - 1} + x_{t + 1} }}{2} $$

For the outliers in the time series of gas concentration, the near average value method is adopted to replace them, and the mean of the two outliers before and after the outliers is taken as the replacement value of the outliers. The outliers before and after processing are shown in Table 2. X_7_ (temperature) was divided into 3000 °C at 11:37 on September 5, 2020, and 24.3 °C was substituted by the near average value method.

**Table 2:** Comparison table before and after outlier processing.

Date	X_1_	X_2_	X_3_	X_4_	X_5_	X_6_	X_7_	X_8_	X_9_	X_10_
2020/9/5 11:15	14.5	0.19	36.72	1300	0.56	1.84	23.7	2649.6	0.05	0.44
2020/9/5 11:37	14.8	0.3	44.56	1165	0.45	2.67	3000	3844.8	0.06	0.16
2020/9/5 12:00	14.8	0.39	31.83	1177	0.51	2.23	24.9	3211.2	0.07	0.2
2020/9/5 12:22	14.7	0.2	25.26	1250	0.34	2.02	24.7	2908.8	0.08	0.27
2020/9/5 12:45	14.6	0.15	43.66	1231	0.62	3.06	23.2	4406.4	0.07	0.13

Date	X_1_	X_2_	X_3_	X_4_	X_5_	X_6_	X_7_	X_8_	X_9_	X_10_
2020/9/5 11:15	14.5	0.19	36.72	1300	0.56	1.84	23.7	2649.6	0.05	0.44
2020/9/5 11:37	14.8	0.3	44.56	1165	0.45	2.67	24.3	3844.8	0.06	0.16
2020/9/5 12:00	14.8	0.39	31.83	1177	0.51	2.23	24.9	3211.2	0.07	0.2
2020/9/5 12:22	14.7	0.2	25.26	1250	0.34	2.02	24.7	2908.8	0.08	0.27
2020/9/5 12:45	14.6	0.15	43.66	1231	0.62	3.06	23.2	4406.4	0.07	0.13

#### Missing value interpolation based on the three-time smoothing index method

There are three strategies for dealing with missing values: directly using data samples with missing values, directly deleting data samples with missing values, and interpolating data samples with missing values. Direct use of samples with missing values reduces the data qualityand the sample variance, and leads to an increase in the prediction accuracy. Direct deletion of samples with missing values omits important feature information, thus affecting the accuracy and accuracy of prediction. Therefore, this paper adopts the strategy of missing value completion.

Methods to complete missing values include: mean interpolation of the samples used, mean interpolation of similar samples, interpolation by modeling prediction method, high-dimensional mapping, compression interpolation, manual interpolation, etc. Since the characteristics in the time series of gas concentration are often affected by many factors, and the factors that affect the time series of gas concentration in the past and the present also determine the development trend in the future, the missing values in the time series of gas concentration are interpolated by the method of cubic smoothing index prediction in this paper.

For missing data within a time period, according to the missing data in the previous period of gas concentration time series value, after determining the length of missing data, data points and smoothing steps are inserted^15,16^.We let the primary smoothing, secondary smoothing and three smoothing steps be $S_{t}^{1}$, $S_{t}^{2}$, and $S_{t}^{3}$, respectively, We take Eq. (2) for processing:

2 $$ \begin{aligned} S_{t}^{1} & = \alpha x_{t} + (1 - \alpha )S_{t - 1}^{1} \\ S_{t}^{2} & = \alpha S_{t}^{1} + (1 - \alpha )S_{t - 1}^{2} \\ S_{t}^{3} & = \alpha S_{t}^{2} + (1 - \alpha )S_{t - 1}^{3} \\ \end{aligned} $$

where α is the weight coefficient of smoothing, m = 3 represents the number of steps, $\widehat{x}_{t + m}$ is the smoothing value of missing data, with $\widehat{x}_{t + m} = a_{t} + b_{t} m + \frac{1}{2}b_{t} m^{2}$; $a_{t}$, $b_{t}$, and $c_{t}$ are the smoothing value coefficients; the running process is shown in Eq. (3); and the comparison of missing values before and after treatment is shown in Table 3, at 1:07 on September 18, 2020. X_8_ (cumulative daily extraction volume) is the missing value, determined to be 5544 by interpolation with the cubic exponential smoothing method^17^.

3 $$ \left\{ \begin{aligned} a_{t} & = 3S_{t}^{1} - 3S_{t}^{2} + S_{t}^{3} \\ b_{t} & = \frac{\alpha }{{2(1 - \alpha )^{2} }}\left[ {(6 - 5\alpha )S_{t}^{1} - (10 - 8\alpha )S_{t}^{2} + (4 - 3\alpha )S_{t}^{3} } \right] \\ c_{t} & = \frac{{\alpha^{2} }}{{(1 - \alpha )^{2} }}\left( {S_{t}^{1} - 2S_{t}^{2} + S_{t}^{3} } \right) \\ \end{aligned} \right. $$

**Table 3:** Comparison table of missing values before and after treatment.

Date	X_1_	X_2_	X_3_	X_4_	X_5_	X_6_	X_7_	X_8_	X_9_	X_10_
2020/9/18 0:22	14.3	0.31	35.37	1300	0.45	2.48	24.8	3571.2	0.07	0.43
2020/9/18 0:45	14.6	0.19	38.91	1250	0.5	3.11	24.8	4478.4	0.08	0.44
2020/9/18 1:07	14.6	0.17	42.76	1235	0.65	3.85	23.6	–	0.09	0.25
2020/9/18 1:30	14.5	0.37	44.28	1246	0.65	3.54	23.5	5097.6	0.08	0.14
2020/9/18 1:52	14.4	0.22	27.94	1282	0.54	1.4	24.3	2016	0.05	0.42

Date	X_1_	X_2_	X_3_	X_4_	X_5_	X_6_	X_7_	X_8_	X_9_	X_10_
2020/9/18 0:22	14.3	0.31	35.37	1300	0.45	2.48	24.8	3571.2	0.07	0.43
2020/9/18 0:45	14.6	0.19	38.91	1250	0.5	3.11	24.8	4478.4	0.08	0.44
2020/9/18 1:07	14.6	0.17	42.76	1235	0.65	3.85	23.6	5544	0.09	0.25
2020/9/18 1:30	14.5	0.37	44.28	1246	0.65	3.54	23.5	5097.6	0.08	0.14
2020/9/18 1:52	14.4	0.22	27.94	1282	0.54	1.4	24.3	2016	0.05	0.42

#### Normalization based on interval scaling

**Figure 3:**
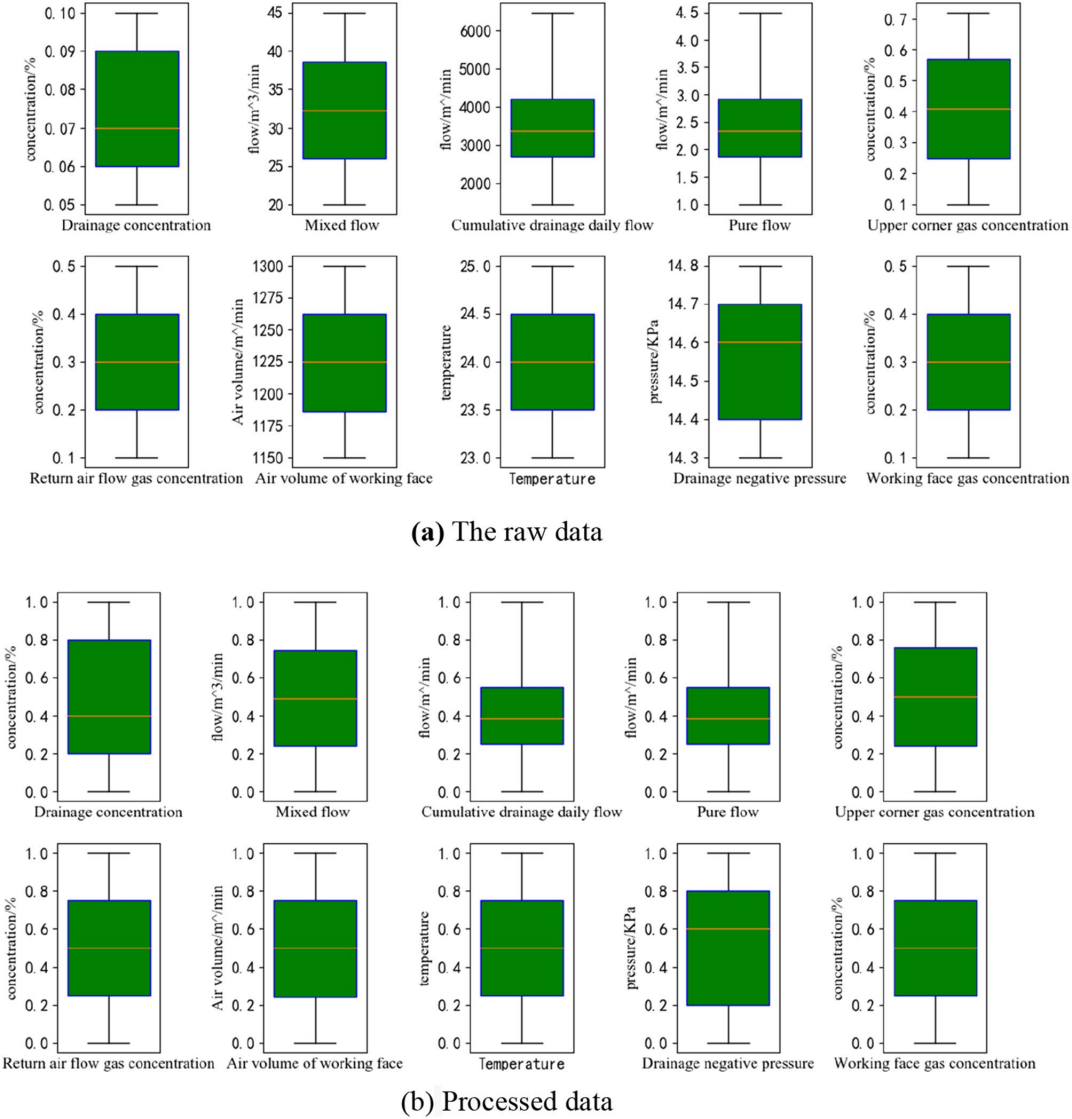
Data presentation.

Since different features have different dimensions and units, to reduce the dimensional influence among features, the data are normalized. In this experiment, the MinMaxScale method was adopted to scale the data uniformly to [0,1]. The method is shown in Eq. (4). where $X_{std}$ is normalized to [0,1], and $X_{Min}$ and $X_{Max}$ represent the minimum and maximum column values,respectively^18^. The raw and processed data are shown in Fig. 3.

4 $$ X_{std} = \frac{{X - X_{Min} }}{{X_{Max} - X_{Min} }} $$

Figure 3a shows the original time series data of gas concentration, and there are different dimensions and units among each feature. To improve the algorithm's convergence speed and prediction accuracy, the data processed by the MinmaxScaler method are shown in Fig. 3b. All features are scaled to [0,1], and there are no dimensional units.

### Construction and optimization of the gas concentration time series feature selection algorithm based on lasso regression

With the development of big data and artificial intelligence, massive and high-dimensional data have become a major challenge in data analysis. There are often many irrelevant variables in feature selection. Failure to effectively remove the irrelevant variables reduces the prediction accuracy. In addition, if important variables are omitted, the model reliability is also reduced. Therefore, feature selection becomes an important step in the model building process. In recent years, the use of coal mine production monitoring data to predict gas concentrations hasmainly focused on time series analysis. This approach usually does not take into account the influence of factors such as temperature, wind speed, negative pressure, and flow, or relied only on manual experience to perform feature selection for the time series of multivariable gas concentrations.

The algorithm used a penalty function to compress the coefficients of the weakly correlated variables to 0 and retained the more relevant strong variables. The purpose of the algorithm was to minimize the impact of the lack of important independent variables on the accuracy of the model and found independent variables with a strong explanatory nature. The algorithm aimed to achieve the effect of feature selection. The core idea of lasso isthat under the constraint that the sum of the absolute values of the regression coefficients is less than a specific constant, the residual error is minimized. Thus a variable with a regression coefficient of 0 can be generated, and a more explanatory model can be obtained. where α is a nonnegative regular parameter,that controls the complexity of the model^19^. The greater the penalty for the linear model, the more features can be incorporated; $\alpha \sum\limits_{i = 1}^{p} {\left| {\beta_{i} } \right|}$ is the penalty item.

5 $$ \beta^{{\prime }} (lasso) = \arg \min^{2} \left\| {\left. {y - \sum\limits_{i = 1}^{p} {x_{i} \beta_{i} } } \right\|} \right.^{2} + \alpha \sum\limits_{i = 1}^{p} {\left| {\beta_{i} } \right|} $$

Lasso uses the L1 absolute value penalty. The regular value of the two-dimensional feature is a quadrilateral. The cost function OLS after L1 regularization is composed of costOLS and costL1, which is equivalent to the contour line and quadrilateral in the figure. The two coordinate axes represent the two features W1 and W2. When the costOLS contour and costL1 intersect at point A for the first time, point A is the minimum value of the cost function. At this time, the feature weighting W2 is equal to 0, thus joining the two features into one. When the number of features rises to hundreds or thousands, costL1 has more corner points. At this time, costOLS intersects the corner point with the highest probability of intersection first, thereby generating a sparse matrix to achieve the purpose of feature selection. In addition, the smaller the penalty coefficient $\alpha$ is, the smaller the regular punishment intensity. The larger the area of the corresponding costL1 is, the smaller the degree of sparse feature weighting. The larger the penalty coefficient is, the greater the penalty intensity and the greater the degree of sparseness^20^.

### Construction and optimization process of the gas concentration time series feature selection algorithm based on lasso regression

**Figure 4:**
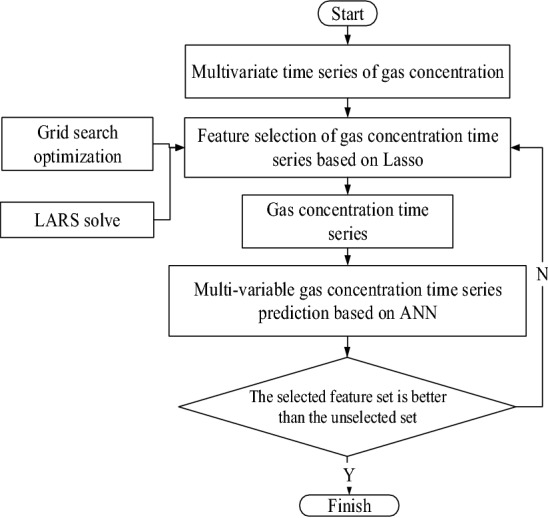
Flow chart of the construction and optimization of the gas concentration time series feature selection algorithm based on lasso.

To further analyze the multivariable gas concentration time series and select the appropriate feature variables, this paper proposes a feature selection algorithm based on the lasso algorithm for the gas concentration time series. Based on the coal mine production monitoring data, the lasso algorithm is used to select the characteristics of the gas concentration time series. The least angel regression (LARS) method is used to optimize the lasso algorithm, and the grid search method is used to determine the optimal value α in the algorithm. The gas concentration time series after feature selection is input, the gas concentration is predicted by the artificial neural network (ANN) algorithm, and a variety of performance indicators are used to evaluate the prediction performance. The detailed lasso feature selection process is shown in Fig. 4.

*Step* 1: α optimization based on grid search

Grid search is an exhaustive search method for specifying parameters. The estimated parameters are optimized through a cross-validation method to obtain the optimal parameter learning algorithm. That is, the possible values of each parameter are freely arranged and combined, and all possible combinations are listed to generate a "grid". The number of grids determines the calculation accuracy and computational cost of the model. Generally, the denser the grid is, the higher the accuracy and the higher the operating cost. In this experiment, we optimize Eq. (5) based on the lasso method and set $\alpha$ as {0.0001, 0.0002,…,10, 20,…,100}.

*Step* 2: Lasso optimization solution based on LARS

LARS is currently the most effective method for solving lasso. LARS also integrates the advantages of the forward gradient algorithm and the forward selection algorithm, which not only retains the accuracy of the forward gradient algorithm, but also simplifies the iterative process of the forward selection algorithm. The calculation process of the LARS algorithm is as follows:

1. Evaluate the relativity of independent variables ${\text{x}}_{{\text{i}}}$ and ${\text{y}}$, and approximate ${\text{y}}$ with the most relevant ${\text{x}}_{{\text{i}}}$;
2. When other variables x and y have the same relativity, that is, when the residuals of the two are the same, the algorithm approaches y along the direction of the "angle bisector" of $x$ and $y$;
3. When the third variable $x_{{\text{k}}}$ has the same correlation with $y$, the algorithm approaches $y$ along the direction of the "angle bisector" common to the three variables;
4. Repeat steps 2–3 until the residual is less than a certain threshold or all the independent variable parameters have been approximated.

**Figure 5:**
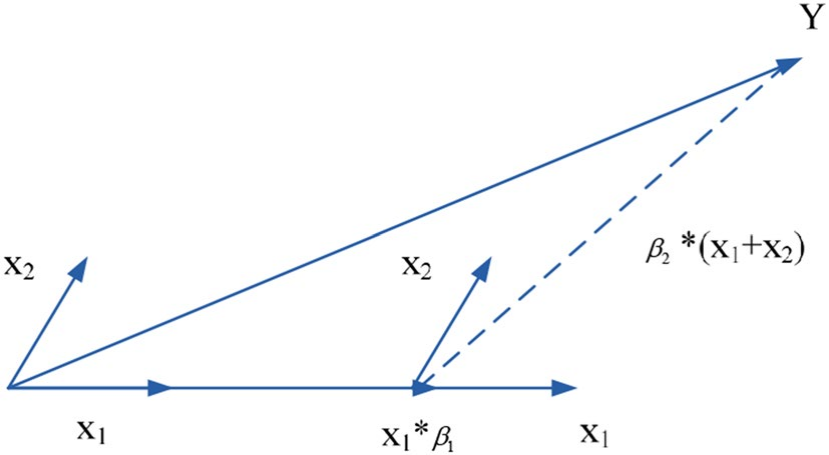
Algorithm solution graph.

A schematic diagram of the LARS algorithm solution is shown in Fig. 5. The correlation between the two independent variables $x_{1}$ and $x_{2}$ and the dependent variable $y$ is $r_{x1} > r_{x2}$, increasing approximately with increasing $x_{1}$ until the residuals of $x_{1} * \beta_{1}$ and $y$ are the same as the correlations of $x_{1}$ and $x_{2}$. That is, the residuals are located in the "corner bisector" of $x_{1}$ and $x_{2}$, approaching along the direction of the $x_{1}$ and $x_{2}$"corner bisectors" thereafter.

*Step* 3: Prediction of gas concentration time series based on the ANN

**Figure 6:**
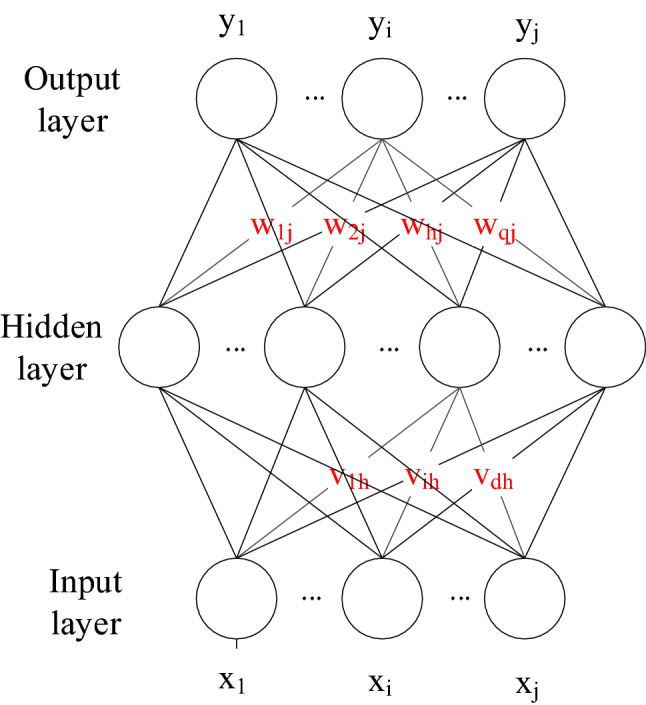
Schematic.

An ANN is a structure that simulates the work of neurons with mathematical models. An ANN includes an input layer, one or more hidden layers, and an output layer. Each layer contains several neurons. The neurons in the same layer are not connected, but the neurons in two adjacent layers are connected^21^. Each element is assigned a weigh; the ANN structure diagram is shown in Fig. 6. Suppose the input is $X = \left( {x_{1} ,x_{2} , \ldots \ldots ,x_{n} } \right)$, the input weight of node h in the hidden layer is $v_{1h} , \ldots ,v_{dh}$ and the corresponding bias is $\gamma_{h}$. The input weight of node j in the output layer is $w_{1j} , \ldots ,w_{qj}$ and the corresponding bias is $\theta_{j}$. The detailed operating principle is as follows:

The input of hidden layer neuron h is:

6 $$ a_{h} = \sum\nolimits_{i = 1}^{d} {v_{ih} x_{i} } $$

The output of hidden layer neuron h is:

7 $$ b_{h} = f(a_{h} + \gamma_{h} ) $$

The input of output neuron j is:

8 $$ \beta_{j} = \sum\nolimits_{h = 1}^{q} {w_{hj} b_{h} } $$

The output of output neuron j is:

9 $$ y_{i} = f(\beta_{j} + \theta_{j} ) $$

*Step* 4: Performance evaluation method based on ANN prediction results

To fully evaluate the prediction performance of the gas concentration time series, performance evaluation indicators were used. The performance evaluation indicators include the regression coefficient of determination (R2), root mean square error (RMSE),and average absolute percentage error (MAPE). The detailed formulas are shows as Eqs. (10)–(12).

10 $$ R^{2} = 1 - \frac{{\sum\limits_{i = 1}^{N} {(y_{i} - E_{i} } )^{2} }}{{\sum\limits_{i = 1}^{N} {(y_{i} - \mathop y\limits^{\_} )^{2} } }} $$

11 $$ RMSE = \sqrt {\frac{{\sum\limits_{i = 1}^{N} {(y_{i} - E_{i} )^{2} } }}{N}} $$

12 $$ MAPE = \frac{1}{n}\sum\limits_{i = 1}^{n} {\left|\frac{{f_{i} - y_{i} }}{{y_{i} }}\right|} *100\% $$

where $y_{i}$ is the true value, $E_{i}$ is the predicted value of $y_{i}$, and $\overline{y}$ is the mean value of the test sample $y$.

### Feature set of time series of working face gas concentration based on grid search

**Figure 7:**
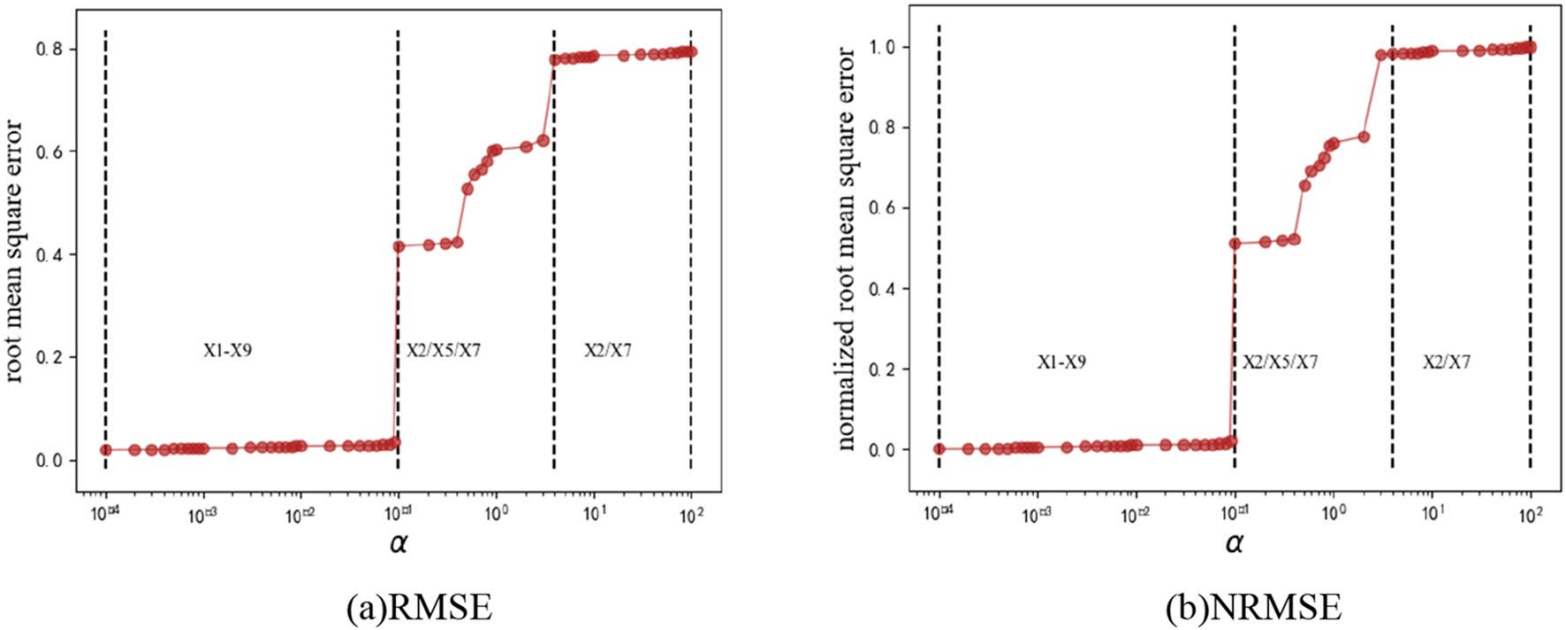
Variable screening.

Based on the processed gas concentration time series, the lasso method is used to select the features of 9 variables. The grid search method is used to optimize the penalty coefficient α in lasso, and we set α ∈ {0.0001, 0.0002,…,10, 20,…100}; the details of lasso variable screening are shown in Fig. 7.

When α ∈ {0.0001,…,0.09}, the RMSE is small, but lasso does not have a special selection effect. At that time, the lasso feature selection results are the return air flow gas concentration (X_2_), upper corner gas concentration (X_5_) and temperature (X_7_). Although it can have a screening effect, the RMSE is too large. Thus this experiment selects 0.2. The result of gas concentration feature selection is the return air flow gas concentration (X_2_), upper corner gas concentration (X_5_) and temperature (X_7_). Similarly, NRMSE changes the value of RMSE to between (0,1).

**Figure 8:**
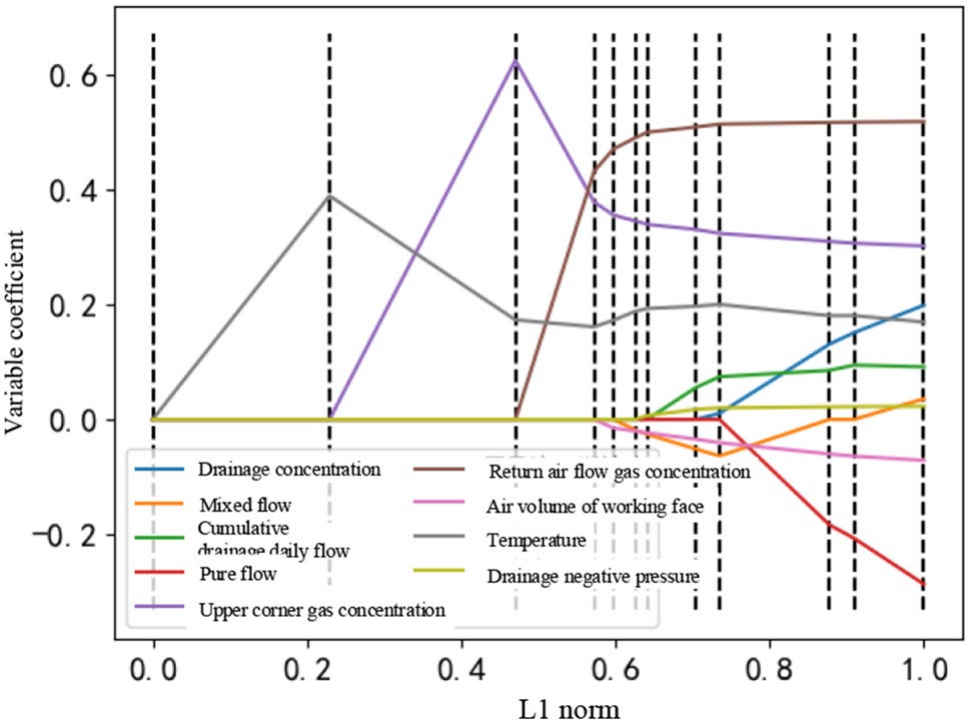
Variable coefficients based on lasso feature selection.

The variable coefficients are used to screen the important parameters that affect the gas concentration of the working face. The coefficients of the screened variables in this article are shown in Fig. 8.

Here, the L1 norm affects the degree of feature sparseness. The larger the corresponding L1 norm is, the smaller the feature and the sparser the feature. Figure 8 shows that the feature selection effect is best when it is 0.2. At this time, L1 = 0.58. At the same time, the pure flow, mixed flow, suction negative pressure, suction concentration, and face air volume are all 0. This indicates that the influence of these 6 parameters on the working face gas concentration is lower than the nonzero value of the three variable coefficients of the upper corner gas concentration, return air flow gas concentration and temperature.

The details of the variable coefficients after lasso feature selection are shown in Table 4.

**Table 4:** Variable coefficients after lasso feature selection.

Variable	Pure flow	Mixing flow	Negative pressure	Upper corner gas concentration	Cumulative drainage daily flow	Return air flow gas concentration	Temperature	Draw concentration	Air volume of the working face	Working face gas concentration
Coefficient	0.0000	0.0000	0.0000	0.4252	0.0000	0.4276	0.1701	0.0000	0.0000	

The upper corner gas concentration coefficient, the return air flow gas concentration and the temperature coefficient are not 0. Therefore, the time series feature set that affects the working face gas concentration selected by the lasso regression algorithm is the return air flow gas concentration, upper corner gas concentration and temperature. The Lasso algorithm uses a penalty function to minimize the residuals under the constraint that the sum of the absolute values of the regression coefficients is less than a specific constant, and compress the coefficients of the weakly correlated variables to 0. Keep the more relevant variables. It achieves the effect of feature selection. It obtains a highly explanatory time series of gas concentration.

## Conclusion

1. The gas concentration time series after feature selection by the lasso method could effectively remove the feature variables. The feature variables were less correlated with the target variable in the gas concentration time series. The gas concentration time series after feature selection by the lasso method could retain the variables with greater correlation with the target variable and improve the explanation of the reliability of the gas concentration time series. The three variables selected by the lasso feature included return air flow gas concentration, upper corner gas concentration and temperature.
2. The lasso compressed feature variables with less correlation through the regular regression method, thereby removing redundant variables. Compared to the gas concentration time series without feature selection, the gas concentration time series after Lasso feature selection could effectively improve the accuracy of the prediction. The input used gas concentration time series after lasso feature selection.

Moreover, the ANN model was used. The prediction performance of the gas concentration time series selected by the Lasso feature as input was more stable. The MAPE could be reduced to 0.3274. The prediction performance was more reliable. The median MAPE was reduced to 0.3625 and R2 was increased to 0.8852.

(3) The experimental results found that the lasso method could obtain a more correlated gas concentration time series. The lasso method used $\alpha$ optimization and the L1 absolute value regularization method for variable screening, which could effectively remove variables that were less correlated with the target variable in the gas concentration time series. At the same time, the prediction model that took the gas concentration time series after lasso feature selection as input could effectively improve the accuracy of gas concentration prediction.
